# Using root metaphors to analyze communication between nurses and patients: a qualitative study

**DOI:** 10.1186/s12909-017-1059-0

**Published:** 2017-11-16

**Authors:** Isabel Álvarez, Laia Selva, José Luis Medina, Salvador Sáez

**Affiliations:** 1 Philosophy and Science of Education, Autonomous University of Barcelona, Barcelona, Spain; 2 Biotechnology. Health Education, University of Lleida, Lleida, Spain; 3 Philosophy and Science of Education, University of Barcelona, Barcelona, Spain; 4 Pedagogy, University of Lleida, Lleida, Spain; 5 Department of Systematic and Social Pedagogy, Autonomous University of Barcelona, 08193 Bellatera, Cerdanyola del Vallès, Barcelona, Spain

**Keywords:** Communication, Metaphors, Worldviews, Chronic patients, Educational health consultations

## Abstract

**Background:**

Metaphors in communication can serve to convey individuals’ backgrounds, contexts, experiences, and worldviews. Metaphors used in a health care setting can help achieve consensual communication in professional–patient relationships. Patients use metaphors to describe symptoms, or how disease affects them. Health professionals draw on shared understanding of such metaphors to better comprehend and meet patient needs, and to communicate information that patients can more easily integrate into their lives.

This study incorporated a theoretical framework based on four worldviews, each with an underlying foundational metaphor (root metaphor). The use of these root metaphors (formism, mechanism, contextualism, and organicism) can have an explanatory function and serve to impart new meanings, as each type of metaphor can lead to a particular interpretation. The study aimed to extract and discuss the root metaphors, with a view to analyzing the communication between health professionals and patients.

**Methods:**

In a case study in Spain over a six-month period, we analyzed the content of recorded, transcribed interviews conducted by one nurse with 32 patients who had chronic illnesses. We inductively extracted five categories that emerged from the interviews: blood sugar, cholesterol, exercise, blood pressure, and diet. We then examined these categories from the standpoint of each of the four root metaphors using two approaches: A series (deductive) and an emergent (inductive) approach.

**Results:**

The results show that the nurse tended to primarily use two worldviews: mechanism and formism. In contrast, patients tended to favor mechanism when discussing cholesterol, blood pressure, and blood sugar levels, whereas contextualism was predominant when the category was diet or exercise.

**Conclusions:**

This study adds to the existing literature on health professionals and patients’ communication. It shows how the use of Pepper’s root metaphors help to analyze the communication between the nurse and patients. Furthermore, it shows they are both using different root metaphors when they are talking about illness and treatments especially regarding blood sugar, cholesterol, exercise, blood pressure, and diet. Further qualitative and quantitative studies are needed to solidly these findings.

**Electronic supplementary material:**

The online version of this article (10.1186/s12909-017-1059-0) contains supplementary material, which is available to authorized users.

## Background

Metaphors are figures of speech that, although not used consciously [1], constitute the roots of human knowledge [2]. Published studies have analyzed the use of metaphors by health professionals and patients [3, 4], in patient explanations of their symptoms [5, 6], and in describing the effects of diseases [7]. Literal metaphors are present in all aspects of the health sector [8].

Most investigations that analyze communication between health professionals and patients stress the importance of metaphors in a positive care relationship [9–12]. Previous studies have focused on the purpose of communication or the analysis of communication in medical and patient-specific behaviors [13–15], the influence of the communication of results to patients [16], verbal and nonverbal communication, the language used by professionals and instrumental and effective communication [17].

There are several factors that can challenge interpersonal communication between nurses and patients because such communication involves unequal positions (with respect to one party having greater knowledge), involuntary relationships, concern issues of vital importance that require close cooperation [15], and in some cases, language barriers [18]. These issues sometimes also involve an inability of the written word to adequately convey complex ideas [19]. Nurses often feel they lack effective communication skills, resulting in a lack of confidence among novice nurses who have recently completed their educational programs [20].

Patients as well as nurses connect new knowledge with lived experiences and weave it into existing narratives of meaning [21, 22]; however, this process is difficult if a person has not had the experience before. The use of metaphors helps to create that narrative process and convey new meanings [1, 23]. Setting up context and providing patients with stories can enable them to embed new ideas (follow new treatments, understanding protocols) and ultimately, to understand and better accept their health condition [21, 24].

Successful self-management of chronically ill patients depends on behavioral changes, which, among other things, in turn depends on effective communication with health professionals [25, 26]. The paradigm of high-quality chronic illness care now seeks to promote a fuller understanding of the patients’ life and preferences [27] and empowering patients [28, 29], helping them with continuous adjustments. In this study, we will show how communication potentially can be more effective if we are aware of the root metaphors and show that knowing and understanding root metaphors could be a starting point for helping chronically ill patients to empower themselves. Chronically ill patients who are thus empowered would, once adequate communication has developed, help clinicians and researchers achieve greater success when a new treatment needs to be introduced.

The aim of this study is to analyze the communication between a nurse and her 32 chronically-ill patients from the standpoint of Stephen Pepper’s root metaphors, as they discuss the treatment and nature the patients’ illness.

### Theoretical framework

In this study, we applied a theoretical framework based on Pepper’s four root metaphors [30] to the emerged categories (blood sugar, cholesterol, exercise, blood pressure, and diet). Pepper distinguishes poetic metaphors from root metaphors. Root metaphors are primarily explanatory and poetic are an important but aesthetic device. This framework is useful with respect to both precision and scope; precision because each root metaphor leads to a way of seeing the truth and reality, to a certain kind of interpretation. Each root metaphor leads to a way of interpreting reality, and each has legitimacy throughout the intellectual history of humankind [31]. Consequently, his work has considerable scope for monitoring issues of meaning and communication [31]. However, working with such a complex theoretical framework requires an abbreviated and workable approach that would give greater practical value to research. An approach of this kind has been elaborated by Kilbourn (see Table 1), who called his distillation of Pepper’s framework an “analytical scheme” [32]. Briefly, these four root metaphors are as follows:

**Table 1:** Adaptation of Kilbourn’s analytical scheme to four worldviews and keywords

	Formism	Mechanism	Organicism	Contextualism
*Root Metaphor*	*Form*	*Machine*	*Organ*	*Context*
	Convention	Cause	Coherence	Change
	Ideal	Efficiency	Connection	Fusion
Keywords	Norm	Frequency	Integration	Intensity
	Plan	Location	Resolution	Relativity
	Similarity	Parts	Synthesis	Vividness
	Tradition	Quantity	Unity	Whole
	How is this item/event similar to other items/events?	How does this item/event work?	How integrated is this item/event?	How intense is the experience of this item/event?

Formism is a worldview whose basis is similarity. The root metaphor of this view is resemblance, comparison or parallelism. On the basis of an intuitive recognition of similarity, the person who holds to formism believes that they can understand things better if they can fit them into a category or specific model. The cognitive process goes from the particular to the general.

The root metaphor of mechanism is the machine. This metaphor is projected as the operation of a mechanism, consisting of large and small pieces which possess autonomy and meaning in their own right, without being a part of the whole to which they belong.

The root metaphor of organicism is integration. It is a hypothesis derived from the recognition that a body is somehow more than the sum of its parts. The basic operation is integrating structures. This worldview is that everything is an organism that lives and moves; organicism is concerned with a sense of process. Its primary source of motivation is from within the individual. Participants are organized and self-regulated in a coordinated and active manner.

The view of contextualism is based on the historical phenomenon. It is never static but is always in perpetual evolution. At the same time, we recognize that if the context changes, so does the event. In this worldview, there are no stable, universal, or exhaustive categories.

In order to become more familiar with how each worldview can help shed different light on a phenomenon, we will use the example of sending an e-mail. Formism focuses on issues of similarity ([33], p. 1358):

“*A formistic orientation on e-mail raises several issues. One is to note that a formist’s focus would be on how an e-mail is similar to and/or different from other forms of communication, particularly written communication. A common comparison is with standard letter writing. E-mail is similar to standard letter writing in that written language is used to convey meaning”*.

Mechanicism stresses issues of causality ([33], p. 1362)*:*

*“Causality operates at different levels. For instance, deep causality involves a thorough theoretical and practical understanding of what happens at the level of computer technology (chip engineering and construction) that enables e-mail systems to exist and messages to be sent and received. Program causality involves having the programming skills and knowledge to be able to develop or troubleshoot an e-mail software program”.*

Organicism’s view of an email would focus on how connected things are ([33], p. 1364):

“*(…) will help to uncover certain ironies about e-mail. Starting with the positive, the connection of people and ideas around the world has increased significantly as a result of e-mail as a medium of communication (…) the ability to connect regularly with people in physically far away places has grown exponentially as a result of e-mail. It has allowed people to be connected and ideas to be exchanged at a volume and pace never before imagined. Political campaigns and protest movements, conducted over the Internet and e-mail, have democratized peoples in ways that could hardly have been anticipated”* (…).

Finally, Contextualism’s view describes the intensity of events ([33], pp. 1360–1361):

“*Booting the computer…Waiting…Clicking the e-mail icon…Scanning the screen…The message?* ***THERE!!!*** *Heart speeding, palms sweating…Pointer moving over the line…deep breath…clickclick…eyes scanning, scanning…Beginning to smile…Reaching for the coffee cup…taking a sip…Reading again, more slow now…Grinning, enjoying…Glancing at the phone, light flashing (did not hear it, call back later). Details go unnoticed in these seamless events. Stepping back, Contextualism allows an appreciation of the nature of the fleeting episode: It is highly fused. It is intense. It is immediate. It is changing. It has threads into the past and future. Its vivid quality is brought out with a contextualist conception of time*”.

## Methods

### Study setting and design

This paper drew on a qualitative interpretative study, specifically using a case study [34, 35] that was conducted over a period of 6 months (from January to June 2014) and involved one nurse’s work with 32 patients who had chronic illnesses. The location of the study was Lleida, Spain. We obtained approval from the Clinical Research Ethical Committee for the entire research period (Ref. P13/071), and written informed consent from patients to participate in the study and for a report to be published using anonymized data.

The setting in which the interviews took place in a local health (public) center was the nurse’s consultation office. This setting was chosen because it was here that the nurse usually spoke to patients, and we did not want to introduce more intrusive elements by transferring them to a different office. This office was located at the end of a corridor, which made it more conducive to recording the interviews, as background noise could be reduced. It was also spacious enough for an observer to be present and thus to take field notes.

We used purposeful sampling to choose the patient group because a bond had to have been created between the nurse and patient due to continuous monitoring over the years; this existing connection would help patients to express their feelings more freely. Purposeful sampling for the nurse was based on her being the only one available who had a significant amount of practice working at the center, and a significant amount of experience teaching at the university, which contributed to her being able to conduct the interviews with each of the patients.

### Participants

There was a total of 32 patients, 18 women and 14 men, and their average age was 71.5 years. The youngest patient was 48 years old and the oldest was aged 85 years. The most prevalent health problems were dyslipidemia, hypertension, diabetes mellitus type 2, and obesity. Other diseases with lower prevalence were ischemic of these pathologies. The overall socioeconomic status of the patient group was middle–low, and most patients had no education beyond the primary level. Only one patient had secondary education, and two were illiterate.

The criteria for the inclusion of nurses were: having a significant amount of professional experience, a nursing consultation office, a significant amount of teaching experience at the university, training in health education, availability, and willingness to participate (this nurse fulfilled these criteria in that she had 25 years of professional experience and 15 years’ experience working at the university, see Additional files 1 and 2). We excluded novice nurses who had no chronically ill patients under their regular care. The criteria for the inclusion of patients were: having received nursing consultation during field research, being a regular patient of this nurse, diagnosis of a chronic disease, and voluntary participation.

### Data collection

The main method used for generating data was face-to-face open interviews with patients. The nurse interviewed each of the 32 patients 3 times. On average, each interview lasted 10–12 min. All interviews were audio-taped and transcribed verbatim. The accuracy of the transcription was checked and compared with the audiotape.

On-site participant observation and field notes also constituted a valuable empirical technique, and served to complement the data drawn from the interviews. Two authors (LS and JLM) alternated, performing five observations each during the first month in order to identify some patterns and help the nurse with the interviews. There were 30 h of observations and both researchers took field notes that served to complement the results and the analysis by describing the communication (or even the silences, and non-verbal communication). The observer was introduced to the patients at the start of each observation session to make it as straightforward and unintrusive as possible. Finally, didactic materials (Textbooks, charts, posters) used by the nurse to convey complex meanings were additional sources of empirical material that served to triangulate the final refinement of the analysis.

### Data analysis

The aim behind the data analysis was to obtain the categories from the interviews, and secondly to identify Pepper’s Root Metaphors in each of the categories.

To begin, after repeatedly reading the transcripts, two researchers (IA and SS) independently coded [36]: the interviews by inductive analysis. This is how five categories emerged (blood sugar, cholesterol, exercise, blood pressure, and diet) out of fifty-five codes that the nurse discussed with patients, by reading and reviewing the entire corpus of collected data. The process of extracting these categories was as follows [37]: (i) all categories were collected; (ii) those that appeared infrequently or only once were removed; (iii) those that appeared most frequently (>10 times altogether) were selected.

Secondly, each category was analyzed following two different but complementary approaches for identifying Pepper’s Root Metaphors (IA and SS). The series approach (deduction) was to analyze each interview from the point of view of each of the root metaphors. For instance, we would take a paragraph of communication and ask how someone would interpret it from a Formist worldview, then from a Mechanist worldview, and so on. This approach was particularly useful when analyzing material that did not suggest any clear reflection of a particular root metaphor. In the emergent approach (induction), the researcher allowed dominant root metaphors to emerge [32] by identifying Root Metaphors’ keywords (Table 1).

To identify Pepper’s four root metaphors, paragraphs were the unit of analysis [36]. The aim in using this scheme in the present study was to make it easier to analyze the transcripts, and to illustrate which root metaphor was reflected in each unit of analysis (see Table 1). Each unit of analysis belonged to one of the emerged (induction) categories, according to what is known [38] as the “comprehensiveness of categories.” However, we also noted that these categories were not mutually exclusive. There were units belonging to more than one category simultaneously.

The final coding system used for the results was as follows. The letters N or P with a number referred to the contribution of either the nurse or the patient, and the interview number; for example, N14 denoted the nurse’s contribution in interview 14 and P9 referred to the patient’s contribution in interview 9. If a letter was not accompanied by a number, it referred to information obtained from the interview. The terms Ca, Cc, Ce, Ct, Cd referred to five categories: blood sugar, cholesterol, exercise, blood pressure, and diet, respectively. Finally, the terms F, M, O, and C referred to the root metaphors formism, mechanism, organicism, and contextualism, respectively. For this last step, we used Kilbourn’s analytical scheme, which illustrated the root metaphors used in each interview. An example will help to understand the coding system used in this report: “*I will take your blood pressure, but this week try to eat salt-free foods*” (N16CdM). This referred to the nurse in interview 16 (N16) speaking about the diet category (Cd) using the root metaphor of mechanism (M) emphasizing Cause-effect component.

## Results

In this section, we will present the results according to each of the five emerging categories that were recorded in the analysis of the nurse’s conversations with patients (table 2).

**Table 2:** Five categories and fifty-five codes

Categories	Blood Pressure	Cholesterol	Blood Sugar	Exercise	Diet
Codes	Cholesterol Exercise Diet Fat Nutrition Walk Arterial hypertension Meal Salt	Blood pressure	Exercise	Cholesterol	Cholesterol
		Exercise	Diet	Blood sugar	Blood sugar
		Diet	Glucose	Diet	Blood pressure
		Fat	Nutrition	Blood pressure	Fat
		Nutrition	Glycated	Fat	Weight
		Walk	hemoglobin	Weight	Glucose
		Arterial hypertension	Walk	Glucose	Nutrition
		Meal	Diabetes mellitus	Nutrition	Walk
			Meal	Walk	Arterial hypertension
			Blood glucose	Arterial hypertension	Diabetes mellitus
				Diabetes mellitus	Meal
				Meal	Salt

The following were examples that illustrated the results in Table 3, representing an overall synthesis of the categories from the interviews. We will start with the “diet” category.

**Table 3:** Synthesis of root metaphors and categories used by the nurse and 32 patients

Nurse (N)	Category	Patients (P)
Formism	DIET (Cd)	Contextualism
Mechanism		
Formism	CHOLESTEROL (Cc)	Mechanism
Mechanism		
Formism	BLOOD SUGAR (Ca)	Mechanism
Mechanism		Organicism
Formism	EXERCISE (Ce)	Contextualism
Mechanism		
Formism	BLOOD PRESSURE (Ct)	Mechanism
Mechanism		Contextualism

### Diet

In this example, the nurse was trying to explain how food is classified according to the food pyramid, and the importance of balancing food intake on a daily basis. The following was an excerpt of their communication, showing how the nurse approached the subject from a Formistic perspective whereas the patient approached it from a Contextualistic perspective:

*N: (…) So in this group we have cereals, pasta … we also have some vegetables, like peas and dried beans, as I already told you. And the other vegetables, along with fruit, make up a different group. From this group, they say that it’s good to eat, between vegetables and fruit, five portions per day” (N27CdF).*

*P: “I eat meat and vegetables every day, but as for vegetables … I cook them in a large saucepan, with lots of onions, carrots …. Then I fill some containers with it and freeze it. (P27CdC).*

Clearly, the nurse was using formism to explain what a healthy diet should consist of on a daily basis. Both concepts were examples of formism because she was using the “*food pyramid*” (the norm), applying healthy habits (the convention) and the balanced diet (the ideal). By contrast, the patient’s answers reflected contextualism when he explained how he did it. He used his experience of many years of making a “*broth*”, where he mixed (fusions) “*vegetables, onions, carrots*”. He never talked about “*food groups”* as a means of (classifying) his balanced diet appropriately, but only his own way of combining them.

### Cholesterol

For the cholesterol category, we have drawn an example in which the nurse maintained a Formistic perspective, whereas the patient adopted a more mechanistic view when it came to explaining that the patient’s cholesterol had risen a bit, according to what the charts (model) indicated. This was the excerpt:

*N: Your cholesterol is also fine, though maybe a little higher than it should be (NICcF).*

*P: “Cholesterol? … But I haven’t eaten any processed food in ages! … Why do I have high cholesterol? I have a sister-in-law who tells me, “take Danacol, they say that it works wonders … sometimes I buy it but I don’t always remember to take it, it’s just another thing I have in the fridge” (P1CcM).*

The patient came in, greeted the nurse effusively. They had known each other for many years. They stared their conversation with high expectations, and when the nurse told him that his cholesterol was “*a little higher than it should be*” the patient frowned, began to get flustered and the expression on his face changed as he asked: *“Cholesterol?”* He placed his right hand on his forehead, a gesture which meant: “I don’t understand!” His attitude had clearly changed. After the consultation was over, and as they said goodbye to each other, he was much less effusive. (Field note U1).

The nurse compared her patient’s cholesterol with the normal levels he should have had and showed it to the patient. The patient was puzzled by the fact that he had abnormal levels when he followed the advice not to eat food that would raise his cholesterol level. He even admitted that he had been taking the kind of functional products recommended by his sister-in-law, which guaranteed lower cholesterol levels. The nurse’s formism appeared when she was comparing (finding similarities) the patient’s cholesterol level to the “standardized charts” (models), which she used to point out to him whether it was low or high. On the other hand, the patient’s mechanistic view was apparent when he referred to the idea of causality *“I have not eaten processed food in ages!”* (the only cause of having a high cholesterol level in the past).

### Blood sugar

The following example was of a conversation between the nurse and one of her diabetic patients. This time the category was blood sugar and showed how the nurse approached it from a Mechanistic view whereas the patient approached it from an Organic view:

*P: I ran nearly two kilometers through the forest and …*

*N: Remember that if you do that you need to take something to eat with you. (N26CaM)*

*P: Sugar?*

*N: If you take sugar your blood sugar levels are going to shoot up, but if that’s what you want to do why not take a sandwich instead? (N26CaM)*

*P: Or I take some glucose, a little bag of dates … (P26CaO)*

*N: But be careful or you might get a scare. (N26CaM)*

*P: No, no. Your body will let you know, then you should stop and rest for a while. (P26CaO).*

The patient added *“running two kilometers”* and the nurse quickly said that if he did that (causality), then he should consider *“bringing something along to nibble”* and the patient said *“sugar”.* She, then, (mechanistically) offered the option of eating a *“sandwich”.* Then she continued with her mechanistic explanation by saying that eating a “*sandwich* (cause) *will not cause his blood sugar level to rise* (the undesired effect), as eating *“plain sugar”* would. The patient’s reaction to the idea of eating a sandwich seemed not to be enthusiastic, and instead suggested a “*bag of dates*”, which tend to be too sugary, so the nurse warned him that that might give him “*a scare”* [dates, like sugar, might give him (cause) a scare (undesired effect)]. The patient showed an organistic approach as he was trying to take some of the advice on board (integrate), but the exchanges were not going very well (connected): e.g. the idea of doing exercise to keep the sugar levels down, but not “*running two kilometers*”, or the idea of eating “*plain sugar”* or “*dates”,* since they are too caloric, so he should replace them with something with fewer calories like a *“sandwich”.* At the end, when the nurse warned him to be careful, he once again adopted an organistic approach by summarizing (synthesizing) the conversation, when he said that *“his body would let him know when he might be tired and needed to rest”* (or, in other words, “*all my body parts are connected in such a way that they will let me know when I have to stop before it is too late*”).

### Exercise

Regarding exercise, the following was an excerpt of their communication conveying the benefits of introducing some exercise into their daily routines. The nurse’s approach was mechanistic and the patient’s was contextualistic.

*N: “You should walk every day. And walking means just that: walking for an hour or half an hour a day, either in the morning or afternoon, whichever you wish. And you shouldn’t say, “today I have a holiday so I’m not going to walk all week and in the weekend I’ll make up for it by walking a lot more and I’ll do some cycling too …” That’s not a good idea. It’s better to do a little every day. Walking three quarters of an hour a day is enough” (N15CeM).*

*P: “Look, I go to the aqua gym, but not when it’s cold outside. Because it tires me out when I go, because they make us work very hard in the water. The main problem is that when I get back home, as I live on the third floor I have to climb 54 steps each time, as many as four times a day (…)” (P15CeC).*

The nurse was explaining that *“walking half an hour or an hour is something the patient should do”*. However, she went on to warn the patient “*not to stop exercising just because she has a week off and then catching up later by doing all the exercise she has not done in a week, since that is not good*”. In other words, according to the nurse, the best way (effective) the exercise could be done was walking: “*walking just three quarters of an hour per day might be enough”* if done on a daily basis, and she even went on to specify the amounts “*an hour, half an hour*” that she should do, the (frequency) “*daily”* and the time of day: “*if you went in the mornings or in the evenings”*. By contrast, the patient responded in a more contextualist manner, when she said she “*stops going to the aqua gym when it is cold outside*”; in other words when there was a (change) in the weather, and she went on to argue that “*she gets very tired and afterwards needs to go up 54 steps to get to her apartment*”. That is to say, her own context came with enough exercise to excuse her from going to the aqua gym. She then explained that there was a level of intensity, when she said “*having to repeat these 54 steps four times a day*!”.

### Blood pressure

Finally, this was an example of helping a patient to understand why her blood pressure had consistently increased. The nurse’s approach was formistic whereas the patient’s was more contextualistic. The nurse used a standardized chart, indicating 140 over 90, and remarked that the patient’s blood pressure should be lower than this. The patient responded, *"Is this higher than him* (my husband)*?”* or *“I’m higher than him”* (P6CtC). This example shows how the nurse used scientific knowledge (formism, by using charts), yet the meaning was clearly not getting across. Instead, when the patient used her husband’s blood pressure values, she was applying contextualism. She had been successful helping to treat her husband’s blood pressure over the years, and had integrated routines for developing healthy habits. Her husband’s blood pressure acted as the “chart” (new model) from which she could understand her own new condition.

We can easily observe from Table 3 that there was no single prevailing worldview; instead, there were several categories which both the nurse and the patients used. Moreover, these categories are not mutually exclusive but rather are interrelated. The table shows a synthesis of the most frequent root metaphors used in the five categories by the nurse and by 32 patients. Mechanism and contextualism predominated among patients in three categories: cholesterol, blood pressure, and blood sugar levels. For the two last categories, diet and exercise, contextualism was prevalent among patients. Diet category proved to be the most difficult to understand, since the nurse approached the matter with formism and mechanism while the patients used contextualism. The nurse did not use contextualism or organicism for any of the categories, with the result that there was less chance of the nurse and the patients understanding each other, especially when the latter use contextualism and organicism (Diet, Blood sugar and Exercise, Blood pressure) as is shown in this example: *“Because the truth is that I have always cared. If I didn’t care, I would have been over 100 kilos. Because I become overweight easily*” (P13CdO).

In our study, the nurse tended to use literal metaphors with didactic intention throughout the educational interventions: “*It is like the tube of a pipe or the wheel of a bike*” (N1), “*The taxi picks up one cholesterol or the other*” (N1), “*Good cholesterol or bad cholesterol is like a snitch (glycosylated hemoglobin) who tells us how the blood sugar has been over the last three months*” (N2), “*Exercise and diet are like stored health; we can always use them in moments of need*” (N). Also, patients used these metaphors to communicate with the nurse: “*I have a large-bone constitution*” (P1). All these expressions are based on metaphors, and some illustrate difficulties in the communication process.

We were able to come up with narratives of meaning through recordings of interviews during nursing consultations. “*The last time you came it was high. It was 7.4%, which is higher than the standard. That was in March. In May, not even three months later, it was 6.5%; it had gone down a lot. That means that things are going well*” (N18). Metaphors are a reflection of this type of interaction within real and specific contexts. The culture in which we are immersed causes us to see reality in a particular way, and the language used, and above all metaphors, are a clear reflection of this. The following is an example of how formism was used to explain cholesterol by comparing good and bad cholesterol types: “*Fats, as we have already said, consist of, among other things, cholesterol. There are two types of cholesterol. There is one type called HDL, which would be the good cholesterol that cares for our arteries, so that they don’t get obstructed. And then, there is the type called LDL or bad cholesterol, which forms blockages inside our arteries that hinder blood circulation*” (N22CcF).

## Discussion

The context within which we conducted this study fell under a very mechanistic and formistic approach, resulting in reducing the complexity of terms such as blood pressure and cholesterol [19]. The cholesterol example showed that the nurse’s formistic approach was inadequate in helping the patient understand the need for adjustments (mechanistically) to his food habits; in fact, it proved ineffective. In this case, the nurse would have done better to use a different approach, and argue that there are other factors (causes) that are currently playing a role in his having a high cholesterol level. However, and despite the fact he was somewhat discouraged by the results of this consultation, the patient needed to be reassured that not eating “*processed food”* was working, but that this should go hand in hand with some other, additional adjustments to his diet. This would contribute to his accepting a more complex set of reasons (causality).

Social, and psychological environments remained in the background and hardly ever arose in the conversations, or at least not as much as the health professional would have liked. We observed a very individualized and personal relationship, with little or no reference to the context of each patient [15]. For instance, within the blood pressure category, if the nurse had used a more contextualistic approach, the patient would probably have understood her better and more quickly; for example, she could have simply said*, “Yes, you are at about the same level as your husband “.* Instead, the nurse insisted and gave the charts (formism) to the woman to familiarize her with this new condition. The use of the same metaphor is more likely to lead to understanding on the patient’s part than an approach that uses two different metaphors.

Discussing illnesses and their symptoms is sometimes challenging and can generate a gap between communication processes (partly owing to the unequal positions with respect to complex knowledge and treatments [39]. Knowledge, analysis, and use of root metaphors can help both the nurse and patient to build bridges, to facilitate communication and understanding of the new stage of a disease [40]. We tell stories, using words to make things easier to understand [41] (as with contextualism). This author [41] described a word as a tool and stories as bridges that no one regrets having created to improve relationships or the communication process. Succinctly put, both professionals and patients need to be allowed to find their own way to create meaning [42].

Disagreements during communication occur because unconsciously we define issues using different root metaphors, as this study has shown (the nurse projects mechanism for explaining the exercise category, while the patient projected contextualism [43]. In this case, the nurse might have benefited from a contextualist approach, making sure the patient could see that going up and down the stairs daily was enough exercise, so she should not feel bad if she stopped going when the weather was “*cold outside”*. The patient talked about a sport she was doing, “aqua gym”. The nurse could have combined both types of exercise: *the stairs and the aqua gym* (fusion), and balanced the amount of exercise needed on a daily basis).

Perceiving these visions or metaphors can help redirect educational interventions and effectively facilitate behavioral change during medical consultations not only among experienced nurses but, more importantly, among novice nurses for whom “communication is huge” [20], affecting their professional confidence. Several studies have drawn attention to certain aspects of the doctor–patient relationship, such as communication and agreement on a diagnosis and treatment, which have a strong association with the resolution of symptoms and better control of hypertension and diabetes [44].

A metaphor needs to be appropriate for the worldview of each patient, those idiosyncrasies that make up their position on the illness–wellness continuum, or the type of language patients use to connect them to their experiences. The key metaphor enables or facilitates communication and understanding. At the same time, the professional needs to adapt to the patient; the professional’s knowledge must facilitate explanation in four alternative ways. Also, professionals can use metaphors to help patients understand a point. Reality is interpreted through our preconceptions, and educational interventions are conditioned by those ideas or thoughts [45]. For instance, diet has different meanings depending on whether our approach to it is formalist (Nurse) or contextualist (Patient). One thing the nurse could have done is to continue the conversation incorporating a contextualist view, by using the example of her patient’s vivid description of making his “*broth”*. She could have asked him what kinds of food groups he normally made besides that broth, and from there she could have pointed out what food groups were missing or not fully represented in his diet. By adapting her patient’s example from the same root metaphor, she could have integrated the new information more appropriately.

In educational health interventions, nurses and patients use different metaphors. In our study, the nurse’s vision was mechanistic and formistic in all categories studied. By contrast, patients used contextualism and mechanism in such categories as cholesterol level, blood pressure, and blood sugar; in the case of the latter, organicism appeared among some patients. With respect to diet and exercise, contextualism predominated (contextualism creates a focus on the emotional aspects of a consultation) [46]. Here, we have discussed a range of different cholesterol levels and diets because, as research shows, different visions of these exist. If the nurse and patients were to use the same vision to convey a category treatment (formism–formism, mechanism–mechanism, contextualism–contextualism and organicism–organicism), the chances of understanding each other could potentially enhance, and thus facilitate, the patient’s treatment. To understand others, it is necessary to stop and listen to their words, stories, and experiences; observe their gestures; describe their contexts, and understand their metaphors (using narratives, [21]; visual material, [19]). The ultimate goal is to integrate the patient’s experience of illness and their values and preferences with information about their medical condition [47]. From our results with blood sugar category, with a nurse approaching it from a mechanistic viewpoint and a patient from a organistic one, the nurse could have taken advantage of her patient’s organistic approach if she had acknowledged that the body indeed lets you know when it is tired, but that requires training and such a level of awareness that sometimes it goes unnoticed, leading to undesirable results. In the context of health education, the visions within which these metaphors emerge must also be examined. There is no single and objective view of reality. Each root metaphor contributes to a slightly different understanding of any concept [32].

Metaphors are present in educational and health care interventions, not only in terms of language, but also thoughts and actions. In other words, they reflect ways of speaking, thinking, doing, and feeling; they show how we perceive reality. It is from experience that patients understand and live; they produce their own metaphors that allow them to explain their experiences in a more personal and meaningful way. We need to provide space for patients to create and be participants in their own metaphors and not be the mere recipients of therapeutic actions, or the metaphors of others. This implies a change of attitude on the part of professionals toward the metaphor of patients as consumers of medical care.

Our research team adopted several strategies to ensure credibility (internal validity), dependability, and confirmability during the data collection and analysis processes [48]. First, to strengthen credibility, we: 1) triangulated methods of data collection and analysis, 2) used a considerable variety of key informants in the study, and 3) used researchers to check and validate whether participants’ root metaphors were adequately interpreted.

### Limitations of the study

This study has some limitations. It was conducted in one city in Spain at a single institution, which may limit transferability to other settings. Additionally, it draws its data from a single nurse and her 32 chronic patients. However, this nurse’s experience can be viewed as an example of a certain group of nurses in a typical nursing consultation. The scope of the study was chronic patients’ recurrent assistance and consultation, focusing on the pre-existing bond between nurse and patients to communicate their health conditions and treatments. We did not interview the other doctors or nurses of this institution. Consequently, we cannot say how the community would apply the framework employed in this study in their daily communication with their patients.

Although this study focused on chronic patients, we presume that the use of root metaphors can be extended to other patients with similar frequently recurring consultations. Our findings add to the existing literature on analyzing patient and health care communication, and we believe they are especially useful to health care practitioners who care for patients with chronic conditions such as mental illness, substance abuse and obesity. Further qualitative studies would solidify these findings, before quantitative studies would be used to investigate the long-term effect of the use of the four root metaphors contributing to what extent these results can be generalized.

## Conclusions

Neither the nurse nor the 32 patients had any former training on Pepper’s framework. However, the use of this theoretical framework unearthed the fact that the nurse and patients were using different (opposing) root metaphors. This increased the chances of discontinuous understanding within the five categories analyzed, especially with regard to the notion of Diet and Exercise, making it difficult for patients to adapt themselves to the changes that the new health condition required, and meant that the positive outcomes did not occur as quickly as some patients might have liked. Finally, as regards the patients’ education, two considerations that might enhance their empowerment could be training health professionals in the use and understanding of root metaphors, and also providing patients with drawings, each drawing illustrating a worldview reflected in common day-to-day situations.

## Additional files

Additional file 1: Letter from the University. (PDF 560 kb)

Additional file 2: Letter from the Ministry of Employment for the nurse. (PDF 137 kb)

## Abbreviations

C: Contextualism.
Ca: Blood Sugar
Cc: Cholesterol
Cd: Diet
Ce: Exercise
Ct: Blood Pressure
F: Formism
M: Mechanism
N: Nurse
O: Organicism
P: Patient
