# Supplementary material for: Using root metaphors to analyze communication between nurses and patients: a qualitative study
Source: BMC Med Educ. 2017 Nov 16;17:216. doi: 10.1186/s12909-017-1059-0 (PMC5689157; doi:10.1186/s12909-017-1059-0)
Supplement: Supplementary file 1 — Letter from the University. (PDF 560 kb) [file 12909_2017_1059_MOESM1_ESM.pdf]

Elimina la filigrana digital ahora

Universitat de Lleida
Servei de Personal
Secció de PDI

Plaça de Víctor Siurana, 1
E 25003 LLEIDA (Catalunya)
Tel. +34 973 70 20 85
Fax +34 973 70 20 87
pdi@personal.udl.cat
http://www.udl.cat/serveis/personal.html

Núm. de orden 4822

Núria Estévez Mur, jefa del Servicio de Personal de la Universidad de Lleida,

CERTIFICO: Que según consta en el Servicio de Personal de esta universidad, la señora [redacted], con DNI [redacted] y NRP [redacted] presta sus servicios como personal laboral, mediante contrato temporal a tiempo parcial, con la categoría de PROFESORA ASOCIADA T1-180H, con actividad asistencial, equivalente a 480 horas anuales de dedicación, de las cuales 180 son de docencia. Está adscrita en el área de conocimiento de Enfermería, del Departamento de Enfermería y Fisioterapia, de la Facultad de Enfermería de esta Universidad.

Que ha desarrollado funciones docentes y/o investigadoras durante los períodos que se detallan a continuación:

- Profesora Asociada 3h (TP)	Universitat de Lleida	De 14/11/1994 a 30/09/1996
- Profesora Asociada T1-3h (TP)	Universitat de Lleida	De 01/10/1996 a 30/09/2008
- Profesora Asociada T1-6h (TP)	Universitat de Lleida	De 01/10/2008 a 05/02/2009
- Profesora Asociada T1-3h (TP)	Universitat de Lleida	De 06/02/2009 a 31/08/2010
- Profesora Asociada T1-90h (TP)	Universitat de Lleida	De 01/09/2010 a 31/08/2011
- Profesora Asociada T1-102h (TP)	Universitat de Lleida	De 01/09/2011 a 31/08/2012
- Profesora Asociada T1-140h (TP)	Universitat de Lleida	De 01/09/2012 a 31/08/2013
- Profesora Asociada T1-180h (TP)	Universitat de Lleida	De 01/09/2013 a

(categoria en la que continua actualmente)

Y, para que así conste y a los efectos oportunos, firmo este certificado a petición de la persona interesada.

pdfelement
