# Supplementary material for: Using root metaphors to analyze communication between nurses and patients: a qualitative study
Source: BMC Med Educ. 2017 Nov 16;17:216. doi: 10.1186/s12909-017-1059-0 (PMC5689157; doi:10.1186/s12909-017-1059-0)
Supplement: Supplementary file 2 — Letter from the Ministry of Employment for the nurse. (PDF 137 kb) [file 12909_2017_1059_MOESM2_ESM.pdf]

GOBIERNO DE ESPAÑA | MINISTERIO DE EMPLEO Y SEGURIDAD SOCIAL

TESORERÍA GENERAL DE LA SEGURIDAD SOCIAL

INFORME DE VIDA LABORAL

De los antecedentes obrantes en la Tesorería General de la Seguridad Social al día [redacted] , resulta que D/Dª [redacted] , nacido/a el 2 de mayo de 1955 , con Número de la Seguridad Social [redacted] , [redacted] , domicilio en [redacted]

ha figurado en situación de alta en el Sistema de la Seguridad Social durante un total de

		60	Años
22.022	**días**	3	meses
		17	días

Presenta las situaciones que se relacionan en las sucesivas hojas del presente informe.

Durante los días indicados en el parrafo anterior Vd. ha estado de forma simultanea en dos o más empresas del mismo Régimen del sistema de la Seguridad Social -pluriempleo-, o en dos, o más Regímenes distintos del citado sistema -pluriactividad-, durante un total de 6.734 días, por lo que el total de días efectivamente computables para las prestaciones económicas del Sistema de la Seguridad Social es de

		41	Años
15.288	**días**	10	meses
		9	días

Cualquier duda o aclaración sobre este informe le será atendida en el teléfono 901 50 20 50, en la web www.seg-social.es o cualquier Administración de la Seguridad Social.

La información sobre las situaciones indicadas no comprende ni los datos relativos a los Regímenes Especiales de los Funcionarios Civiles del Estado, de las Fuerzas Armadas y de los Funcionarios al servicio de la Administración de Justicia, ni los datos relativos a los períodos trabajados en el extranjero.

A los efectos previstos en el artículo 5 de la Ley Orgánica 15/1999, de 13 de diciembre, de protección de datos de carácter personal se informa que los datos incorporados en el presente informe se encuentran incluidos en el Fichero General de Afiliación, creado por Orden de 27 de julio de 1994. Respecto a los citados datos podrá ejercitar los derechos de acceso, rectificación y cancelación en los términos previstos en dicha Ley Orgánica.

INFORME DE VIDA LABORAL
Situaciones

DATOS IDENTIFICATIVOS

NOMBRE Y APELLIDOS	Nº SEGURIDAD SOCIAL	DOCUMENTO IDENTIFICATIVO
[redacted]	[redacted]	[redacted]

SITUACIÓN/ES

RÉGIMEN	EMPRESA SITUACIÓN ASIMILADA A LA DE ALTA		FECHA ALTA	FECHA DE EFECTO DE ALTA	FECHA DE BAJA	C.T.	CTP %	G.C.	DÍAS
GENERAL	25004186958	INSTITUT CATALA DE LA SALUT	15.12.2000	15.12.2000	---	---	---	02	6.041
GENERAL	25103782680	UNIVERSITAT DE LLEIDA	01.09.2016	01.09.2016	---	501	28,8	01	87
GENERAL	25004186958	PLURIEMPLEO. DISTRIBUCION DE TOPES	01.09.2016	01.09.2016	30.09.2016	---	---	--	
GENERAL	25004186958	PLURIEMPLEO. DISTRIBUCION DE TOPES	01.11.2013	01.11.2013	31.08.2016	---	---	--	
GENERAL	25004186958	PLURIEMPLEO. DISTRIBUCION DE TOPES	01.10.2016	01.10.2016	---	---	---	--	
GENERAL	25103782680	UNIVERSITAT DE LLEIDA	01.09.2015	01.09.2015	31.08.2016	501	29,3	01	107
GENERAL	25103782680	UNIVERSITAT DE LLEIDA	01.09.2014	01.09.2014	31.08.2015	501	29,3	01	107
GENERAL	25103782680	UNIVERSITAT DE LLEIDA	01.09.2013	01.09.2013	31.08.2014	501	29,3	01	107
GENERAL	25103782680	UNIVERSITAT DE LLEIDA	01.09.2012	01.09.2012	31.08.2013	501	22,7	01	83
GENERAL	25103782680	UNIVERSITAT DE LLEIDA	01.09.2011	01.09.2011	31.08.2012	501	16,5	01	60
GENERAL	25103782680	UNIVERSITAT DE LLEIDA	01.09.2010	01.09.2010	31.08.2011	501	14,6	01	53
GENERAL	25103782680	UNIVERSITAT DE LLEIDA	01.10.2009	01.10.2009	31.08.2010	501	14,6	01	49
GENERAL	25103782680	UNIVERSITAT DE LLEIDA	06.02.2009	06.02.2009	30.09.2009	501	14,6	01	35
GENERAL	25103782680	UNIVERSITAT DE LLEIDA	01.10.2008	01.10.2008	05.02.2009	501	29,2	01	37
GENERAL	25100156496	UNIVERSITAT DE LLEIDA	01.10.2003	01.10.2003	30.09.2008	518	16,0	01	292
GENERAL	25100156496	UNIVERSITAT DE LLEIDA	14.11.1994	14.11.1994	30.09.2003	---	---	01	3.243
GENERAL	25004186958	INSTITUT CATALA DE LA SALUT	01.05.1991	01.05.1991	13.12.2000	---	---	02	3.515
GENERAL	25004362063	FORMEMP, S.L.	17.10.2000	17.10.2000	29.11.2000	004	20,0	01	9
GENERAL	25004747033	ADIFRUIT, S.L.	01.03.1990	07.03.1990	31.10.1994	---	---	01	1.700
GENERAL	25000829546	I.C.S. HOSPITAL ARNAU DE VILANOVA	29.03.1990	05.04.1990	30.04.1991	---	---	02	391
GENERAL	25000829546	I.C.S. HOSPITAL ARNAU DE VILANOVA	01.02.1990	01.02.1990	27.03.1990	---	---	02	55
GENERAL	25000829546	I.C.S. HOSPITAL ARNAU DE VILANOVA	01.03.1989	01.03.1989	30.01.1990	---	---	02	336
GENERAL	25000829546	I.C.S. HOSPITAL ARNAU DE VILANOVA	20.09.1985	20.09.1985	31.01.1989	---	---	02	1.230
GENERAL	25000829546	I.C.S. HOSPITAL ARNAU DE VILANOVA	01.03.1976	01.03.1976	08.03.1978	---	---	02	738
GENERAL	25002750954	I.C.S. HOSPITAL ARNAU DE VILANOVA	30.01.1976	30.01.1976	29.02.1976	---	---	02	31
GENERAL	25002750954	I.C.S. HOSPITAL ARNAU DE VILANOVA	15.07.1975	15.07.1975	04.08.1975	---	---	02	21
GENERAL	25000	MUNPAL DIP PROV LLEIDA	01.02.1979	01.02.1979	10.09.1985	---	---	--	2.414
GENERAL	25000	MUNPAL DIP PROV LLEIDA	01.01.1979	01.01.1979	31.01.1979	---	---	--	31
GENERAL	25000	MUNPAL DIP PROV LLEIDA	01.08.1975	01.08.1975	31.12.1978	---	---	--	1.249

Este documento no será válido sin la referencia electrónica. La autenticidad de este documento puede ser comprobada mediante el Código Electrónico de Autenticidad en la Sede Electrónica de la Seguridad Social, a través del Servicio de Verificación de Integridad de Documentos.

INFORME DE VIDA LABORAL

Notas aclaratorias

Los informes de vida laboral contienen información respecto de las situaciones de alta o baja de una persona en el conjunto de los distintos regímenes del sistema de la Seguridad Social. Las situaciones que se incluyen en los informes son computables para el acceso, al menos, de una de las prestaciones contributivas del sistema de la Seguridad Social. Por lo tanto, no todas las situaciones que se incluyen en el informe de vida laboral tienen que ser necesariamente computables para todas las prestaciones económicas contributivas del sistema, aspecto éste que deberá ser determinado por la Entidad Gestora competente sobre la resolución de la solicitud de la correspondiente prestación.

A continuación se aclaran algunos conceptos y denominaciones usados en el informe de vida laboral que pueden ayudarle a comprender el contenido del mismo. No todos los conceptos que se detallan tienen que aparecer necesariamente en su informe de vida laboral dado que alguna de las denominaciones son específicas de determinados Regímenes o situaciones concretas.

RÉGIMEN

Identifica al Régimen en el cual se encuadra el correspondiente periodo. Puede ser alguno de los siguientes: Régimen GENERAL, Régimen Especial de Trabajadores por Cuenta Propia o AUTÓNOMOS, Régimen Especial AGRARIO, Régimen Especial de los trabajadores del MAR, Régimen Especial de la minería del CARBÓN o Régimen Especial de empleados de HOGAR. Dentro del Régimen GENERAL se identifica al colectivo de REPRESENTANTES DE COMERCIO, y al SISTEMA ESPECIAL DE FRUTAS, HORTALIZAS Y CONSERVAS VEGETALES.

EMPRESA

Se consigna el código de cuenta de cotización o número de inscripción del empresario utilizado para la individualización de éste en el respectivo Régimen del Sistema de la Seguridad Social, así como la denominación de la empresa u organismo a cuyo nombre figura el código de cuenta de cotización.

SITUACIÓN ASIMILADA A LA DE ALTA

Situación diferente a la de la prestación de servicios o actividad determinante de su encuadramiento en un régimen del sistema de la Seguridad Social, que surte efectos respecto de las prestaciones, contingencias y en las condiciones que para cada una de ellas se establecen en el Reglamento General sobre inscripción de empresas y afiliación, altas, bajas y variaciones de datos de trabajadores aprobado por el Real Decreto 84/1996, de 26 de enero, y en las demás normas reguladoras de las mismas.

FECHA DE ALTA

Fecha de inicio de la prestación de servicios o de la actividad, o fecha de inicio de la situación asimilada a la de alta.

FECHA DE EFECTO DEL ALTA

Fecha a partir de la cual tiene efectos el alta en orden a causar derecho a las prestaciones del sistema de Seguridad Social, salvo para las prestaciones derivadas de accidentes de trabajo y enfermedades profesionales, desempleo y asistencia sanitaria derivada de enfermedad común, maternidad y accidente no laboral, en las cuales la fecha de efecto de alta coincide, en cualquier caso, con la fecha de alta.

FECHA DE BAJA

Fecha de cese de la prestación de servicios o de la actividad, o fecha de cese de la situación asimilada a la de alta.

FECHA DE EFECTO DE BAJA

Fecha a partir de la cual se extingue la obligación de cotizar.

CONTRATO DE TRABAJO (C.T.)

Clave que identifica a efectos de la gestión de la Seguridad Social, la modalidad del contrato de trabajo. (*)

C.T.P.-% SOBRE LA JORNADA HABITUAL DE LA EMPRESA

En los contratos de trabajo a tiempo parcial el coeficiente, en tantos por ciento, identifica el porcentaje que, sobre la jornada a tiempo completo establecida en el Convenio Colectivo de aplicación o, en su defecto, sobre la jornada ordinaria máxima legal, realiza o ha realizado el trabajador/a. (*)

GRUPO DE COTIZACIÓN

Grupo de categorías profesionales en el que se incluye al trabajador/a. (*)

BASE DE COTIZACIÓN

Base de Cotización por la que se ha optado en el Régimen Especial de los trabajadores por Cuenta propia o Autónomos o en determinados convenios especiales. En situaciones de alta, la Base de Cotización es la que consta en la fecha de emisión del informe. (*)

Este documento no será válido sin la referencia electrónica. La autenticidad de este documento puede ser comprobada mediante el Código Electrónico de Autenticidad en la Sede Electrónica de la Seguridad Social, a través del Servicio de Verificación de Integridad de Documentos.

INFORME DE VIDA LABORAL

Notas aclaratorias

MEJORA DE I.T.

En el Régimen Especial de Trabajadores por Cuenta Propia o Autónomos y en el Régimen Especial Agrario, determina si el trabajador ha optado por tener cubierta la prestación económica de incapacidad temporal. (*)

ENTIDAD DE I.T.

En el Régimen Especial de Trabajadores por Cuenta Propia o Autónomos y en el Régimen Especial Agrario, identifica la Entidad Gestora de la Seguridad Social o Mutua de Accidentes de Trabajo y Enfermedades Profesionales con la que se ha formalizado la cobertura de la incapacidad temporal. (*)

ENTIDAD DE A.T. Y E.P.

Identifica la Entidad Gestora de la Seguridad Social o Mutua de Accidentes de Trabajo y Enfermedades Profesionales con la que se ha formalizado la cobertura de los riesgos profesionales.(*)

COEFICIENTE DE PERMANENCIAS EN EL SISTEMA ESPECIAL DE FRUTAS, HORTALIZAS Y CONSERVAS VEGETALES

Coeficiente multiplicador a aplicar a los días efectivamente trabajados en el sistema especial.

DIAS DE TRABAJO EN EL SISTEMA ESPECIAL DE FRUTAS, HORTALIZAS Y CONSERVAS VEGETALES

Número de días efectivamente trabajados en el sistema especial.

DIAS A LOS QUE NO ES DE APLICACIÓN EL COEFICIENTE DE PERMANECIAS EN EL SISTEMA ESPECIAL DE FRUTAS, HORTALIZAS Y CONSERVAS VEGETALES

Número de días en situación de alta en el sistema especial a los que no resulta de aplicación el coeficiente de permanencias.

DIAS EN ALTA

Número de días comprendidos entre la FECHA DE EFECTO DEL ALTA y la FECHA DE BAJA. En situaciones de alta el número de días se computa entre la FECHA DE EFECTO DEL ALTA y la FECHA DE EMISIÓN DEL INFORME.

PECULIARIDADES DE LOS CONTRATOS A TIEMPO PARCIAL:

Al número resultante de la diferencia entre la FECHA DE EFECTO DEL ALTA y la FECHA DE BAJA se ha aplicado el porcentaje sobre la jornada habitual de la empresa. En el supuesto de que en un período el trabajador haya tenido distintas jornadas de trabajo en cuanto a su duración, en el cálculo de los días se han tenido en cuenta todas ellas. El cálculo del número de días en situación de alta en períodos con contrato a tiempo parcial es provisional. El cálculo definitivo de los días teóricos de cotización se efectuará en función del número de horas ordinarias y complementarias efectivamente trabajadas. Este cálculo se realizará en el momento de que se efectúe una solicitud para el acceso a una prestación económica del sistema de la Seguridad Social. En cualquier caso, para las prestaciones de jubilación e incapacidad permanente, al número de días en situación de alta se le aplicará el coeficiente multiplicador de 1.5.

PECULIARIDADES DEL CONVENIO ESPECIAL DE FUNCIONARIOS DE LA UNIÓN EUROPEA

Los días en situación de alta de este convenio especial únicamente son computables para el acceso a la prestación económica de incapacidad permanente derivada de contingencias comunes.

PECULIARIDADES DEL SISTEMA ESPECIAL DE FRUTAS, HORTALIZAS Y CONSERVAS VEGETALES

Si en el informe constan los datos de COEFICIENTE DE PERMANENCIAS, DÍAS DE TRABAJO y/o DÍAS A LOS QUE NO ES DE APLICACIÓN EL COEFICIENTE DE PERMANENCIAS el número de días en situación de alta se calcula multiplicando los DÍAS DE TRABAJO por el COEFICIENTE DE PERMANENCIAS, sumándose al resultado obtenido los DÍAS A LOS QUE NO ES DE APLICACIÓN EL COEFICIENTE DE PERMANENCIAS.

La situación de alta de forma simultánea en dos, o más, Regímenes distintos del citado sistema -pluriactividad-, siendo una de las empresas del Sistema Especial de Frutas, Hortalizas y Conservas Vegetales, impide determinar si al número de días calculado según se ha indicado en el párrafo anterior se le deben restar días por existir una superposición de períodos cotizados. El cálculo definitivo se realizará en el momento en que se efectúe una solicitud para el acceso a una prestación económica del sistema de la Seguridad Social.

(*) En el supuesto de que el trabajador, en cada período, haya tenido más de un contrato de trabajo, porcentaje sobre la jornada habitual de la empresa, grupo de cotización, base de cotización, mejora incapacidad temporal y/o entidad que cubre la prestación de incapacidad temporal, sólo aparece en el informe de vida laboral el último de cualquiera de estos datos.
